# Estrogen modulation of pain perception with a novel 17β-estradiol pretreatment regime in ovariectomized rats

**DOI:** 10.1186/s13293-019-0271-5

**Published:** 2020-01-09

**Authors:** Wenxin Zhang, Hui Wu, Qi Xu, Sheng Chen, Lihong Sun, Cuicui Jiao, Luyang Wang, Feng Fu, Ying Feng, Xiaowei Qian, Xinzhong Chen

**Affiliations:** 1 Department of Anesthesia, Women’s Hospital, Zhejiang University School of Medicine, Xueshi Road 1, Hangzhou, 310006 China; 2 Zhejiang University School of Medicine, 866th Yuhangtang Road, Hangzhou, 310058 China

**Keywords:** Estrogen, Nociception, Hyperalgesia, Estrogen receptors

## Abstract

Estrogen plays substantial roles in pain modulation; however, studies concerning sex hormones and nociception often yield confusing results. The discrepancy could be a result of lack of consensus to regard estrogen as a variable when working with animal models; thus, the influence of hormones’ fluctuations on nociception has continually been neglected. In the present study, we designed a novel hormone substitution model to aid us to evaluate the effects of estrogen’s long-term alterations on ovariectomy (OVX)-induced mechanical hyperalgesia and the expression of estrogen receptors(ERs). OVX rats were implanted with slow-release estrogen pellets at differently arranged time points and doses, such that a gradual elevation or decrease of serum estrogen levels following a relatively stable period of estrogen replacement was achieved in rats. Our results demonstrated that gradual estrogen depletion rather than elevation following the stable period of estrogen substitution in OVX rats alleviated OVX-induced mechanical hyperalgesia in a dose-independent manner, and the opposite estrogen increase or decrease paradigms differently regulate the expression of spinal ERs. Specifically, in rats rendered to continuously increased serum estrogen, the early phase estrogen-induced anti-nociception effect in OVX rats was eliminated, which was accompanied by an over-activation of ERα and a strong depression of ERβ, while in the OVX rats subject to gradual decrease of estrogen replacement, both ERα and ERβ increased modestly compared with the OVX group. Thus, the present study demonstrated that estrogen increase or decrease modulate nociception differently through change of spinal ERs.

## Introduction

It is commonly recognized that females manifest different pain sensitivity under physiological and pathological conditions compared with males, and numerous chronic pain syndromes tend to aggravate during the premenopausal and ovulation periods. Estrogen is perceived to be a fundamental contributor to the sex-based disparities in nociceptive responses. However, previous studies yielded confusing results and both pro- and antinociceptive effects of estrogen have been observed. A tempting explanation for these contradictions might be that most of the studies investigated only the causal relationship between the presence or absence of estrogen and the consequent behavioral or biological responses. However, the continuous increase or decrease of estrogen that was also important in pain modulation has been neglected.

The ever-changing characteristic of estrogen, as is manifested by its fluctuation during the menstrual cycle and peri-menopause periods, makes it extremely difficult to reveal the mechanisms underlying its pain modulatory effects during diverse physiological and pathological conditions. The majority of animal studies endeavored to investigate estrogen’s effects on nociception had adopted the gonadectomy and hormone replacement modalities, and by this means, they simplified the essential clinical settings into a paradigm in which only the presence or absence of estrogen is considered, and thus failed to treat estrogen as a variable factor. Consequently, the related studies have yet to reach a consensus result on the pain modulatory effects of estrogen. It has been established that OVX rodents developed obvious hyperalgesia and allodynia that could last for as long as 4 months [1], and numerous studies favored an analgesic effect of estrogen on OVX-induced hypersensitivity in intact or inflamed animals [2–8]. However, other literatures mentioned conflicting reports. It has been reported that OVX rats showed a significantly less nociceptive response during the interphase of formalin inflammatory pain, which could be inversely modulated by exogenous estrogen [5, 9, 10], and estrogen itself was capable of producing hyperalgesia in intact and castrated rats and quails [11, 12]. Apart from the confusing results derived from behavioral studies, considerable efforts have been devoted to clarify the link between pain modulation by estrogen and the quantitative changes of estrogen receptors. OVX was reported to either downregulate or upregulate estrogen receptors in both brain regions and the spinal cord [13–18]. Concurrently, the auto-regulation of ERs by their ligand has also been extensively studied. While short-term estrogen treatment was reported to differently tune the expression of the receptor subtypes ERα and ERβ [19, 20], chronic estrogen substitution was suggested to decrease the expression of both ERs [14, 21].

The massive contradictions have long been attributed to different timing and dose of estrogen replacement, as well as the tissue of interest and pain modalities examined. However, in retrospect of previous literatures, we noticed that the chronic increase or decrease of estrogen probably weigh far more than the other factors in pain modulation. A pharmacokinetic study by Ito et al. lends further support for this hypothesis [22]. They demonstrated that the universally applied slow-release pellets (Innovate Research, USA) could not maintain a steady systemic estrogen level as was claimed by most studies, in fact the serum estrogen concentration continued to decrease to a significantly lower level compared with that at the time of implantation; thus, it was essentially upon this chronic hormone withdrawal states that most of previous studies based their conclusions. In light of the results, several more complex models were proposed to explore the influence of different estrogen paradigms on neuronal plasticity. Studies indicated that short- or long-term estrogen administration differently determined its analgesic effects [22–24]. Markowska et al. showed that chronic estrogen replacement only when primed with repeated injections was effective in restoration of cognition [25]. However, to our knowledge, none has yet employed a rigorous strategy to investigate the effects of dynamic estrogen alterations on pain regulation. It should be noted that the current studies could not exclude the effects of other hormones and various in the modulation of pain perception, and thus, the results should be interpreted carefully when put into the clinical contexts.

In light of the above observations, we hypothesized that a long-term estrogen change, either with a gradual elevation or decrease in system level, would differently affect pain perception and expression of estrogen receptors. In the present study, we trialed with a bi-directional replacement paradigm to achieve either gradual elevation or decrease of serum estrogen concentration during estrogen replacement in OVX rats, and observed the effects of dynamic estrogen alterations on OVX-induced mechanical hyperalgesia and changes of spinal ER expressions.

## Materials and methods

### Subjects

Forty adult virgin female Sprague–Dawley rats at the age of 3 months were used. The animals were housed four per cage in a humidity-controlled room with free access to food and water, the temperature of the facility was maintained at 22 °C with a 12:12-light-dark cycle. The rats were acclimated to manipulation every day during the 7-day-adaptation period to reduce the stress induced by the environment. Rats went through OVX or pellet implantation surgery were housed individually for 7 days and then reformed with their groups. All experimental procedures were carried out in accordance with the National Institute of Health Guide for the Care and Use of Laboratory Animals (NIH Publications No. 86-23). The Animal Ethics and Welfare Committee of Zhejiang University School of Medicine approved all the experimental protocols. An effort was made to minimize the number of animals used and their suffering.

#### Ovariectomy and estrogen replacement

Rats were randomly divided into five groups, intact (*n* = 6), sham (*n* = 6), OVX (*n* = 10), E-decrease group (*n* = 8), and E-increase group (*n* = 5). Rats in all groups went through OVX surgery except for the sham group.

**Fig. 1:**
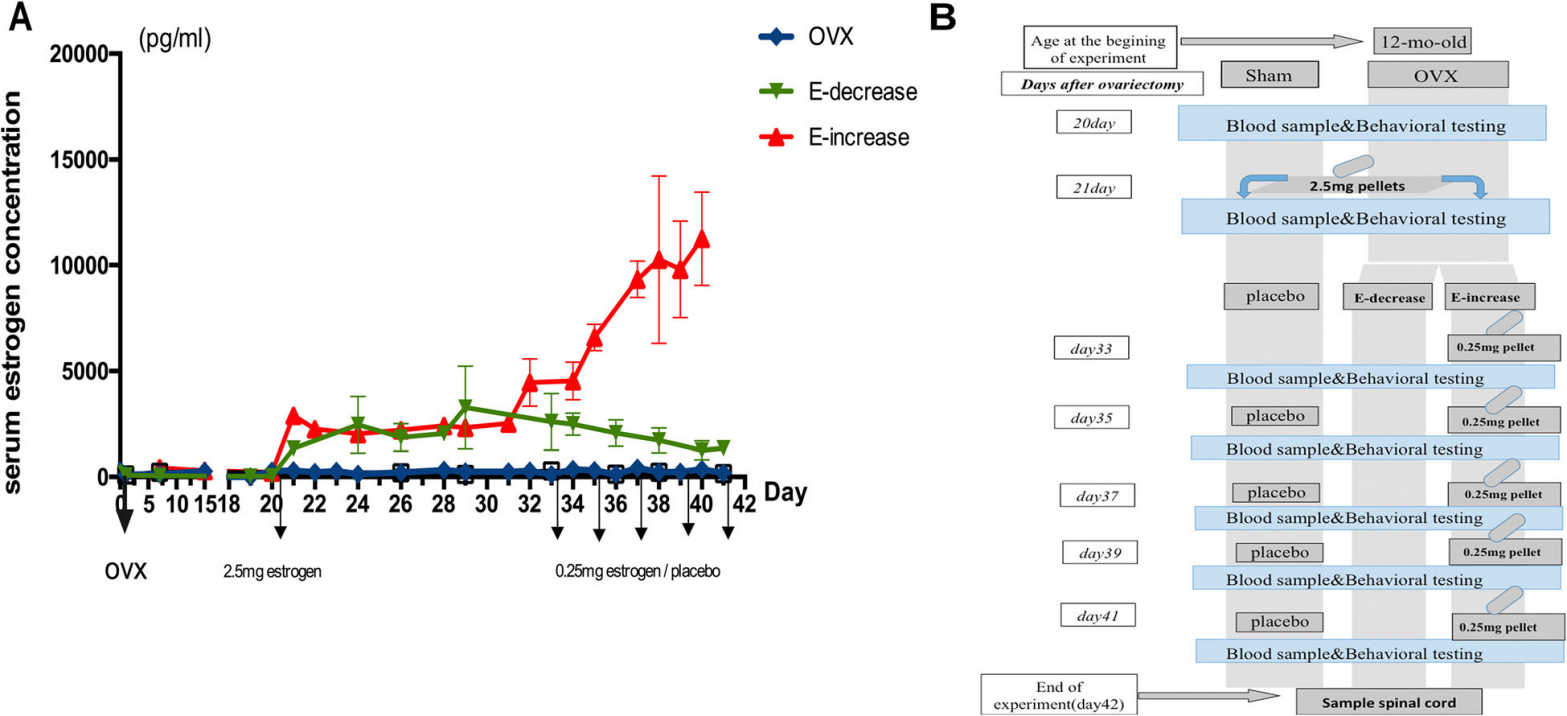
Establishment of different estrogen pretreatment regimes. **a** Fluctuations of serum estrogen levels in rats with different replacement paradigms. The day of OVX surgery was defined as day 0, and arrows indicate the day of estrogen pellets implantation. From day 33 on, the groups showed different estrogen alteration trends. Data are showed as mean ± SEM. **b** Illustration of the substitution paradigms: control (intact) group was omitted from the diagrams for the convenient of comprehension

After a wash-out period of 20 days after OVX, the rats were implanted with slow-release estrogen pellets or placebo pellets, and the day of OVX surgery was defined as day 0. All animals underwent surgeries with 1.5% isoflurane (HeBeiYiPin, China) in oxygen through a nose mask for anesthesia. Briefly, in rats subjected to estrogen replacement, the estrogen pellets or placebo were implanted subscapularly into a skin pocket gently made with the forceps in rats anesthetized with isoflurane, and the pellets were implanted with manufacturer’s apparatus (Precision Trochar, Innovate Research of America, US) to minimize the injury. Specifically, the rats in E-decrease group were implanted with one single 2.5-mg estrogen pellet on day 20, and rats in the E-increase group were implanted with one 2.5-mg pellet on day 20, and then administered with 0.25-mg pellets on every morning of days 33, 35, 37, 39, and 41; at the same time, the rats in E-decrease group were also implanted with the 0.25-mg placebos with the same time frame as in the E-increase group (Fig. 1b). In this way, every 48 h, a 0.25-mg pellet was added, as this interval time was calculated according to the manufacturer’s data sheet regarding the drug’s half-life and in vivo elimination rate, as well as our verification of the specific metabolite timescale of the pellets (Additional file 4: Figure S3). As a result, the replacement paradigm of the E-decrease and E-increase group was established such that a continuous increase or decrease of serum estrogen trend could be achieved in OVX rats. The rats in the sham group were administered with placebo pellets from the same manufacturer with the same temporal pattern as the E-increase group. All rats were weighted every five days during the experiment.

The slow-release estrogen pellets were purchased from the Innovate Research Company (Innovate Research, US) as the one used in most of previous studies. The temporal and dosing of the replacement of drugs was based on our pilot pharmacokinetic experiments results. Only one kind of drug (slow-release pellet) and delivery method was used in the experiments in order to eliminate any influence of pharmacokinetic variance induced by drug form and delivery routes.

#### Vaginal cytology

To monitor the stages of estrus cycle and the effects of estrogen manipulation, vaginal smear of rats were taken daily before the blood extraction and behavioral tests to record the estrous stages at the time of tests. In experiments involving estrogen pellets implantation, vaginal smears were taken before the surgery. A cotton tip dipped with 0.9% sterile saline was inserted into the vaginal and scrubbed gently, then daubed on a microscope slides. The smears were stained with methane blue and different stages of the estrous cycle were determined using cytology methods according to the description by Freeman (1994). In brief, the pro-estrous was differentiated by a predominance of large, round, nucleated epithelial cells, and an absence of leukocytes, estrus is identified by a majority of cornified squamous epithelial cells, densely distributed leukocytes indicate di-estrous, and met estrus is identified by scattered cells of all three types.

#### Blood sample and hormone levels quantification

Blood was sampled every 2 days for animals in all the groups. Under isoflurane anesthesia, 1~1.5-ml blood was collected to a 1.5-ml centrifuge tube from the tail vein of rats, and 1 ml of saline was given to the animal immediately after the blood collection for supplementation of the fluid loss. What’s more, during the experiment, all the animals had normal fluid intake and kept gaining weight steadily according to our observations. The blood was allowed to coagulate at room temperature for 2 h and then centrifuged at 5000 rpm for 10 min at − 4 °C. After centrifugation, the supernatant was separated into another clean centrifuge tube and frozen at – 20 °C for later detection. Estrogen and corticosterone concentration were measured by high-performance liquid chromatography (HPLC). An HPLC system (aglient1290-AB 5500MSD, Agilent Technologies, Calif., USA) was used for chromatographic separation and evaluation, and an equipped software of the HPLC system named Analyst Software was used to record and confirm the spectra peaks in all samples.

#### Testing of mechanical nociceptive threshold

Rats in all groups were subjected to electronic von Frey test daily. The von Frey test apparatus (Ugo Basile, Comerio, Italy) consists of three acrylic boxes (23 × 18 × 14 cm, each) with a mesh floor through which it was possible to stimulate the plantar with a needle connected to a mechanosensor. The apparatus recorded the paw withdrawal thresholds (PWT) automatically, and it was more accurate and efficient in measuring mechanical sensitivity compared with the traditional von Frey hand-held filaments. The behavioral tests were always taken at 10:00 am to reduce any variation related to circadian rhythm. Experiments were conducted in a dark and noise-free room; after a 20-min acclimation, a linearly increasing mechanical force was applied to the rat’s dorsal hind paw with the metal von Frey filament. The stimulation force was applied manually in accordance to the synchronized curve provided by the software on the computer. PWL was defined as the force at which the rat withdrew its paw abruptly. Each rat was tested 5 times the most with a 2-min separation, and the three most nearest results with a difference of no more than 10 mN were adopted and averaged. Care was taken not to stimulate the same point of hind paw twice to avoid learning or hypersensitivity and a 20-s cut time was set. A well-trained investigator blind to the group assignment conducted all tests. Spontaneous behaviors (exploratory duration, rearing frequency, and grooming duration) were recorded for 60 min once a week before the von Frey tests.

#### Immunohistochemistry

At the last day of the experiments, rats were deeply anesthetized with isoflurane and perfused intracardially with 200~300 ml 4% formaldehyde in phosphate-buffered saline (PBS). The lumbar (L4~L6) spinal cord was removed and then post-fixed for 4 h in 4% paraformaldehyde, cryoprotected overnight in 30% sucrose in PBS at 4 °C. The spinal cord was then serially sectioned (8 μm thick) in a transverse plane and mounted on microscope slides. For immunohistochemistry, the sections were stained using avidin–biotin–peroxidase complex technique. Sections were incubated with normal goat serum (Boster, WuHan, China) + 0.3% Triton-X for 1 h at room temperature to block non-specific staining. Slides were then incubated overnight with primary ER antibodies (rabbit anti-ERα polyclonal antibody, 1:100, Abnova, China; rabbit anti-ERβ polyclonal antibody, 1:50, Sigma, USA) in 5% bovine serum at 4 °C. After washing in PBS, slides were incubated with biotinylated goat anti-rabbit IgG (1:400, Boster, WuHan, China) from the manufacture’s kit for 30 min at room temperature, washed in PBS, and treated with avidin-biotin reagent for 30 min at room temperature. After three washes in PBS, immunostaining was revealed with 1% diaminobenzidine tetrahydrochloride, 0.3% H_2_O_2_ and 1% nickel chloride in PBS (Boster, WuHan, China). Slides were observed under a microscope and reactions stopped by flushing under tap water when a brown background appeared (4 min). Slides were then counterstained with hematoxylin (Beyotime, Shanghai, China) for 1 min and terminated by washes with tap water. Slides were finally dehydrated through graded ethanol solutions followed by xylene and then cover-slipped with neutral balsam (Aladdin, Shanghai, China). The ERα and ERβ primary antibodies were validated with western blot experiment (Additional file 4: Figure S3b). For immunofluorescence, the GPR30 primary antibody (1:500, rabbit anti-GPR30 polyclonal antibody, Abcam, Hong Kong) was used. Slides were then incubated with FITC-conjugated goat anti-rabbit secondary antibody (1:400, Beyotime, Shanghai, China) for 30 min at room temperature and stained with 4,6-diamino-2-phenyl indole(DAPI) for nuclei staining. In the control experiments, tissues were processed using the same staining protocol, but omitting the primary antibodies. For quantification of the immunolabeling results, coverslips were viewed using a fluorescence microscope (Eclipse Ni-E, Nikon, Shanghai, China). Images were analyzed using the Image J version 1.50i (National Institute of Health, USA). For quantification of ER positive neurons, slices were taken from each of L4~L6 spinal cord segments of the groups. The optical density of ER positive neurons was analyzed in lamina I and II of the spinal cord. Under a × 40 microscopic field of the slides, a consistent threshold was set on all photographs. After subtracting the background threshold, the software calculated the average optical density of the area of interest. A value of the optical density divided by the number of nuclei in the same area was deemed as the inmmunoactivity of the slides.

#### Statistics

All results were presented as mean ± SEM. The difference of estrogen levels and PWT measures at different time points among groups were analyzed using two-way ANOVA, with Bonferroni’s multiple comparisons test for pairwise comparisons. The semi-quantified data of immunohistochemistry of ER expression in each group were compared using 2-tail paired Student *t* test. Pearson’s correlation coefficient was used to test for correlations among the different hormonal levels and PWT measures. Statistical analysis was performed using SPSS Statistics Version 22.0 (IBM Corporation, Armonk, NY). *P* < 0.05 was considered statistically significant.

## Results

### Establishing a gradual increase or decrease of serum estrogen levels in OVX rats

Two weeks after ovariectomy, the serum levels of estrogen declined to a very low level, which is close to the minimum detectable by the quantitative assay (10 pg/ml), and this level lasted throughout the experimental range (Fig. 1a). It should be noted that the estrogen levels in both placebo and intact group fluctuated within a normal range (20 pg/ml~150 pg/ml), and were thus not shown in the results. The estrogen treatment paradigms in our experiments differently altered serum estrogen levels. In the E-decrease group, the serum estrogen level was raised in the first 3 days after a single estrogen pellet implantation and remained relatively constant for the next 6 days before a gradual yet significantly decline was observed. At the last day (day 41), the serum concentration in E-decrease group was 1350 pg/ml on average, 98.3% of that at the initiation of the substitution (day 20). On the ninth day of the substitution when the estrogen level was the highest in the E-decrease group, the average concentration was 3280 pg/ml and 238.84% of that at the initial replacement (day 20).

As for the E-increase group, from the 11th day of the replacement, 0.25-mg pellets were added every 48 h, and this paradigm induced continues acceleration of serum estrogen concentration throughout the later phase of the replacement term. The highest estrogen concentration of the E-increase group was reached at the end of the experiment at 11255 pg/ml and almost eight times higher than that of the E-decrease group. According to pharmacokinetic observations in our pilot study, a single 0.25-mg pellet implanted in OVX rats would result in a rapid serum estrogen surge that peaked at 24 h after implantation and lasted for at least 2 days, and then it decreased rapidly to approximately physiological serum level at day 21 (Additional file 2: Figure S1). Thus, 48 h was supposed to be the approximate half-life of the 0.25-mg drug pellet under the current replacement condition. Our results demonstrated that with a carefully designed replacement strategy, both chronic increase and decease of estrogen levels in OVX rats within a 21-day frame could be achieved. This model could serve as a tool for our investigation of the effects of bi-directional hormone changes on pain modulation.

Considering the potential effects of multiple blood collection on the HPA axis and animal’s stress levels, we tested the serum corticosterone concentration as well as evaluated the grooming durations of animals in different groups. Our results showed that the blood extraction procedure did not have significant effects on rat’s corticosterone levels when compared with their initial levels during the experiment (Additional file 3: Figure S2). In addition, the behavioral results showed that the grooming duration was higher in OVX compared with the control group, which was reduced by estrogen replacement (Additional file 1: Table S1). Taken together, these results further confirmed that the multiple blood collection method did not have significant stressful influence on the animals in the experimental regime.

### Estrogen decline or elevation during estrogen replacement differently modulates mechanical hypersensitivity in OVX rats

**Fig. 2:**
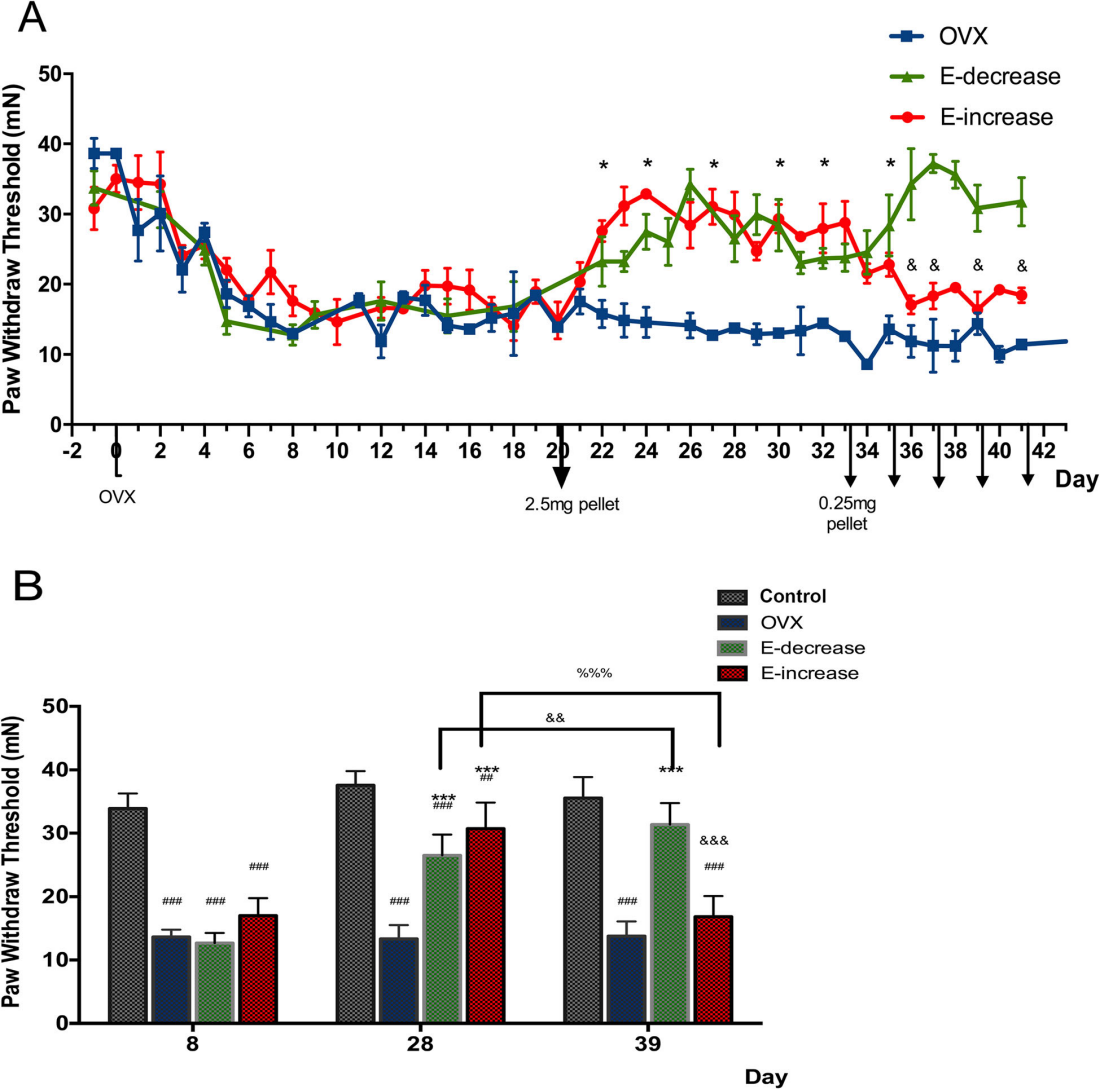
Behavioral changes to different estrogen pretreatment regimes. **a** Changes of paw withdraw threshold with date in groups of OVX (blue), E-decrease (green) and E-increase (red) rats. The day of OVX surgery was defined as day 0, and the mechanical nociception tests started 2 days prior to OVX and lasted until day 41. Arrows indicate the doze and time of estrogen pellets implanted. 2.5-mg pellets were applied on day 20 to both E-increase and E-decrease group, and 0.25-mg pellets were applied in E-increase group only on day 33, 35, 37, 39, and 41 respectably. Data are shown as mean paw withdraw threshold (± SD) to three times of electronic von Frey stimuli. *P < 0.05 when compared with the OVX group, ^&^*P* < 0.05 when compared with the E-decrease group. Significant differences (one-way ANOVA with Bonferroni’s multiple comparison test; *P* < 0.05) were detected in both estrogen replacement groups compared with the OVX group started from day 22 until the end of the experiment. And the PWT of the E-increase group significantly attenuated (one-way ANOVA with Bonferroni’s multiple comparison test; *P* < 0.01) starting from day 36 compared with that of the E-decrease group. **b** Grouped histographs show PWT difference among different groups at selected time points as day 8, day 28, and day 39 after OVX. Data are shown as mean paw withdraw threshold (± SD) to electronic von Frey stimuli. ^#^*P* < 0.05 when compared with the control group, *P < 0.05 when compared with the OVX group, ^&^*P* < 0.05 when compared with the E-decrease group, and ^%^*P* < 0.05 compared with the E-increase group

ANOVA analysis revealed evidently different trends in the alterations of the PWT among different estrogen replacement groups. We did not observed any significant changes of the PWT in female rats at different phases of estrous cycle in a previous study (data not shown), the PWT falls within 33~38 mN as were observed in our experiments. Our results demonstrated that OVX rats developed a significant mechanical hyperalgesia (Fig. 2a), the PWT progressively decreased starting from 1 week after the OVX surgery to as low as 40.3% of the control level, and this hyperalgesia state in rats lasted until the end of our experiment. In the E-decrease group where rats were replaced with a single 2.5-mg pellet on day 20, the OVX-induced hyperalgesia was reversed immediately 2 days after estrogen implantation. Their PWT measures recovered gradually to a point approximate to the level before the ovariectomy at the end of the replacement session (day 41). This result matches with those of previous studies proving estrogen’s anti-hyperalgesia effects. Two-way ANOVA revealed that during the early phase (from day 20 to day 33) of estrogen replacement, the pain threshold in the E-decrease group increased rapidly, and was then maintained at a relatively steady state, while during the later phase of the replacement (from day 33 to day 41), the PWT increased even more dramatically compared with that of the early phase (Fig. 2b).

**Fig. 3:**
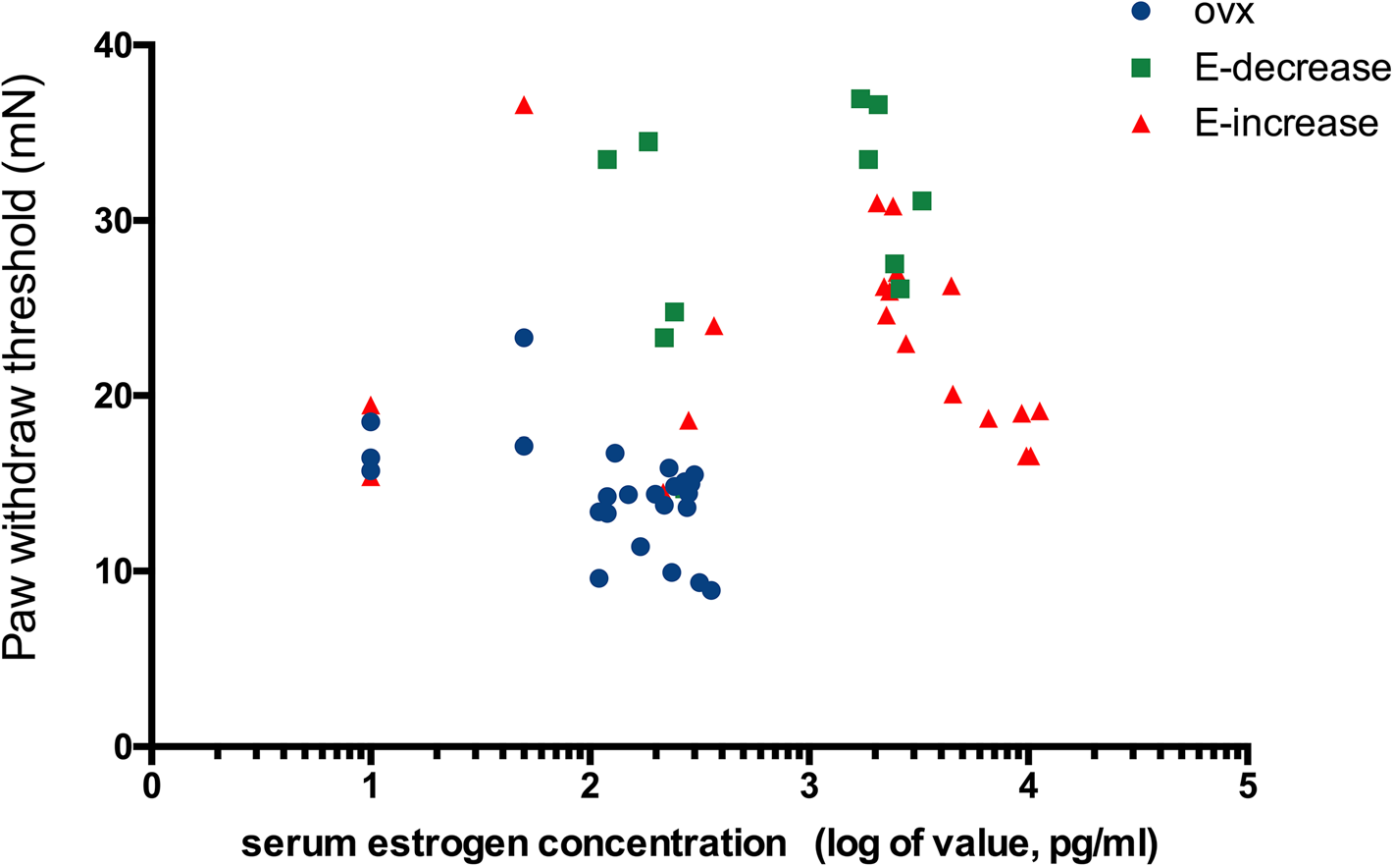
Correlation between serum estrogen and PWT. Analysis of correlation between serum estrogen levels and the PWT value of each group (Pearson’s correlation coefficient). Data are shown as mean ± SD

Concurrently, the E-increase group also reversed the OVX-induced mechanical hyperalgesia during the early phase of the 2.5-mg pellet replacement as was with the E-decrease group. Surprisingly, starting from day 34 when the first 0.25-mg pellet is implanted, the PWT in the E-increase group took a decreasing trend and this downregulation continued with the other 4 0.25-mg pellets implanted in succession. At the end of the experiment (day 41), the PWT in the E-increase group was significantly lower than that in the E-decrease group (57.9%), and maintained at a level close to that in the OVX rats (Fig. 2b). In addition, during the entire range of the experiment, the E-increase group did not show pain threshold significantly higher than that of the E-decrease group at any time point, despite the fact that their serum estrogen concentration has been significantly higher than the E-decrease group. Collectively, these results demonstrated that (1) estrogen replacement reversed the OVX-induced hyperalgesia in a dose-independent manner and (2) the late phase estrogen decline or elevation during estrogen replacement differently modulated mechanical hypersensitivity, as the late phase estrogen decrease fully reversed the OVX-induced mechanical hyperalgesia, while the late phase estrogen increase inflicted an contrary effect and abolished the early phase estrogenic analgesia. Furthermore, to investigate if there is a correlation between the serum estrogen concentrations and the PWT values, we conducted Pearson’s correlation analysis. The resulting Pearson’s correlation coefficient revealed a negative correlation between the PWT and estradiol serum levels in the OVX group (*n* = 24, *r* = − 0.53, *P* = 0.0083). However, there were no significant correlations between the PWT and estrogen levels in the E-decrease and the E-increase groups during the estrogen replacement phase (day 20~day 41). The results suggested that estrogen levels at a given time point was not correlated with the mechanical nociception under the current estrogen substitution circumstances (Fig. 3). In addition, a single 0.25-mg estrogen replacement also attenuated OVX-induced hyperalgesia with a rapid reduced serum estrogen levels starting from pellet implantation (Additional 4: Figure S3).

Estrogen decline or elevation during estrogen replacement differently modulates estrogen receptor expression

**Fig. 4:**
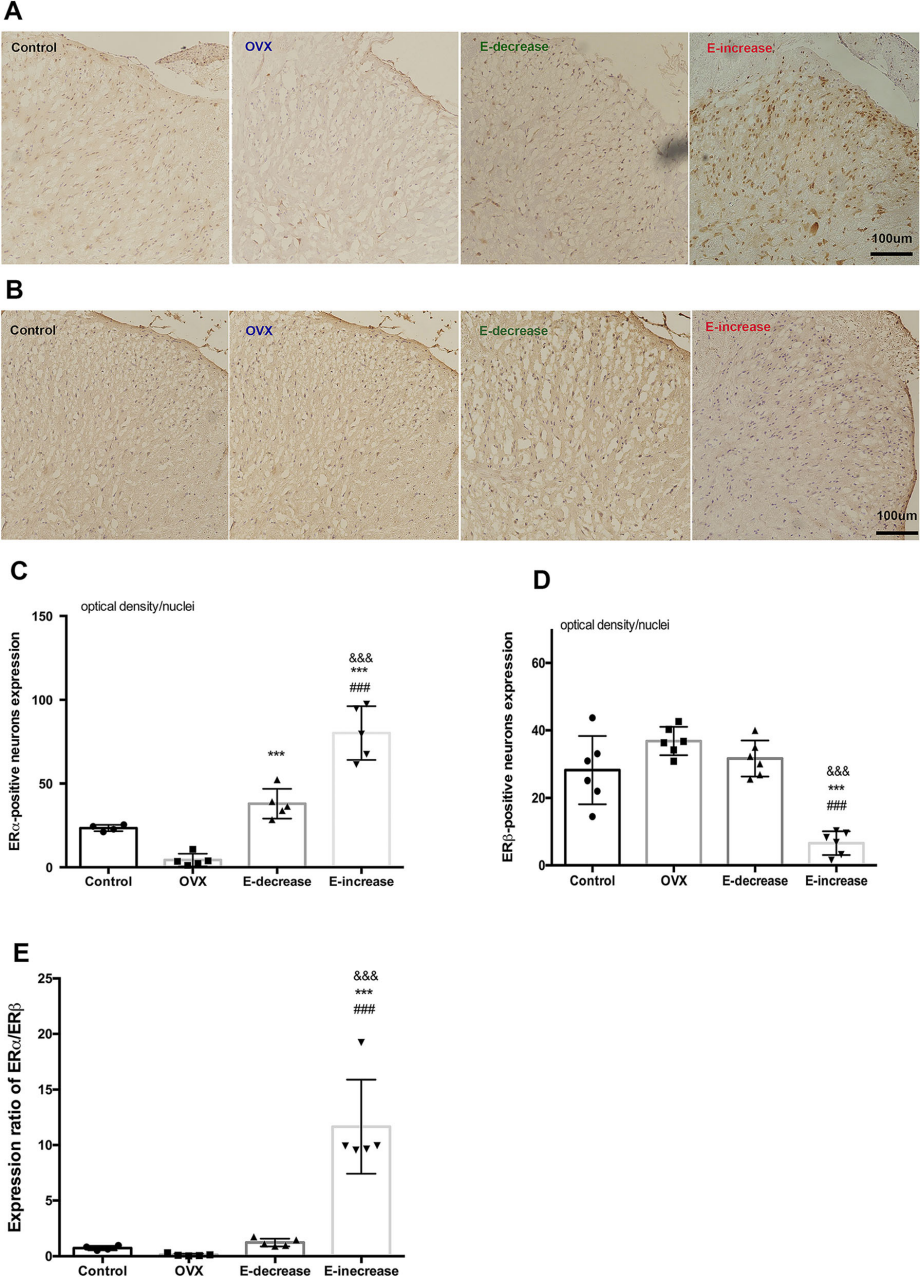
Expression change of ERα and ERβ to different estrogen pretreatment regimes. Expression of **a** ERα and **b** ERβ positive neurons in rat spinal cord at the last day of estrogen substitution. ER positive neurons mainly concentrated in the laminae I~II of the spinal cord, pictures are shown at × 20 magnification, scale bar represents 100 μm. **c**, **d** Histographs show one-way ANOVA analysis of the difference of ER expression among groups. Data are shown as mean ± SD. *P < 0.05 when compared with the OVX group, ^&^*P* < 0.05 when compared with the E-decrease group. **e** Expression ratio of ERα to ERβ of each group

**Fig. 5:**
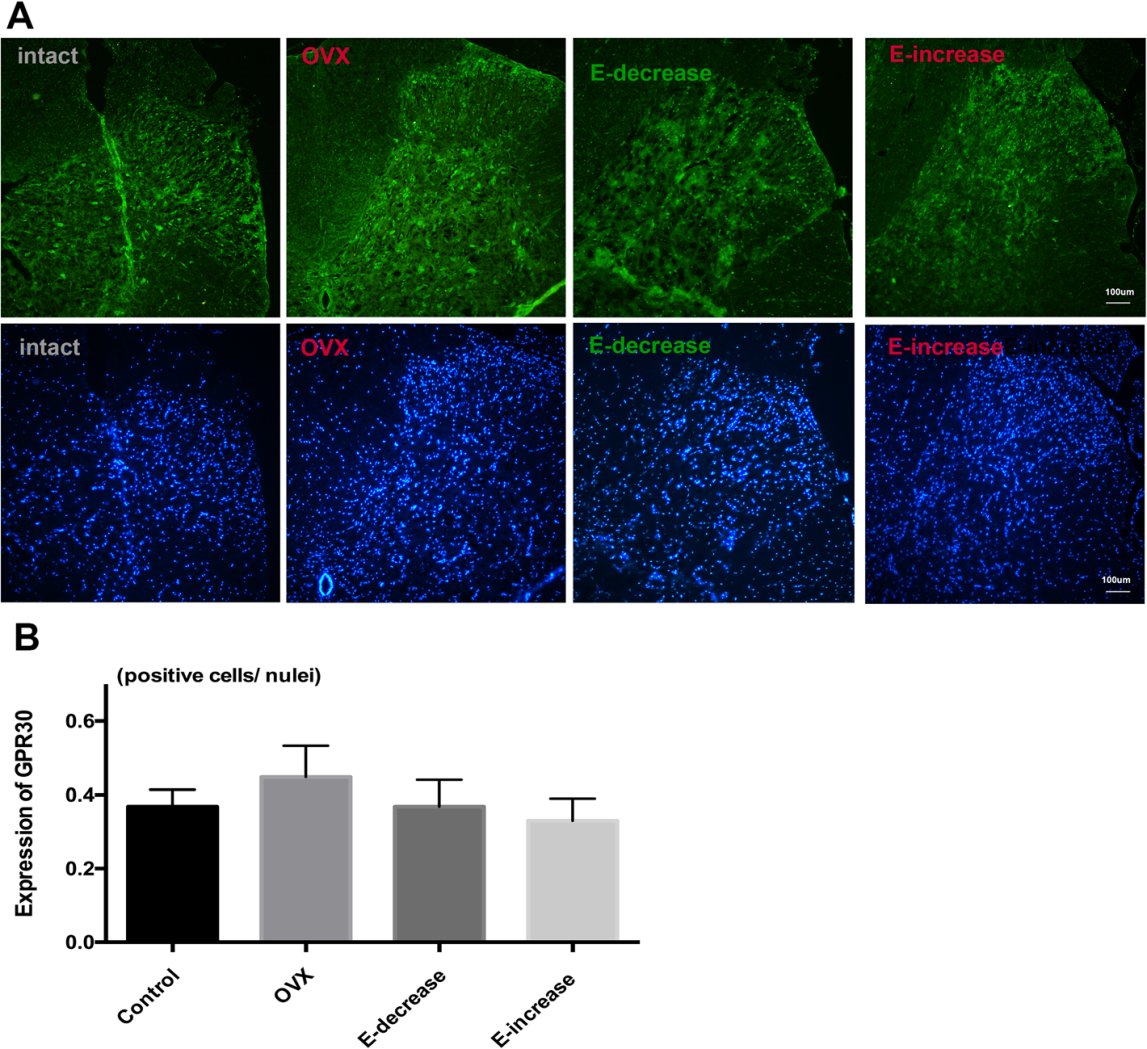
Change of expression of GPR30 under different estrogen pretreatment regimes. **a** GPR30 positive neurons in the spinal cord in rats of different groups at the end of the experiment. Pictures were shown at x 10 magnification. Scale bar represents 100 μm. **b** Histographs show one-way ANOVA analysis of the difference of GPR30 expression among groups. Data are shown as mean ± SD, and no significance was detected among the groups

At the end of measuring the PWT, the expression of estrogen receptors in the spinal cord was evaluated and compared among different groups. It should be noted that at the lumbar spinal cord of rats, ERs were mainly distributed in lamina I~II, which was verified in our results (Fig. 4). ERα showed a more predominant expression than ERβ in control sham-operated rats. In comparison with the control group, long-term OVX significantly reduced the expression of ERα (Fig. 4a, c, *P* < 0.05), while ERβ was not affected significantly (Fig. 4b, d). These results were in agreement with a previous study [16], which reported ovariectomy decreased ERα but not ERβ mRNA levels in rat spinal cord. Post hoc comparison revealed that estrogen replacement in E-decrease group significantly increased ERα expression compared with the OVX group (*P* < 0.001), while the expression of ERβ was hardly influenced (Fig. 4c, d). Interestingly, in E-increase group, the results showed that the late phase estrogen surge dramatically upregulated the expression of ERα compared with all the other groups (*P* < 0.001 vs. sham, OVX, E-decrease group); simultaneously, the expression of ERβ was strongly depressed in the spinal cord at the last day of the replacement. To view the change more clearly, the expression ratio of the two receptors was compared among different groups, and the results showed that rats in the E-increase group manifested the largest ERα/ERβ ratio (Fig. 4e). Furthermore, we also evaluated the expression of membrane estrogen receptor GPR30, and the results showed that different estrogen fluctuation modes did not inflict significant effects on GPR30 protein expression (Fig. 5). Negative controls omitting the first antibodies resulted in no positive stains (Additional file 5: Figure S4), and all the observations of ERs positive stains were conducted on the L4~L6 spinal cord segments (Additional file 6: Figure S5).

### The effects of estrogen replacement on vaginal smear and weight change

OVX induced a di-estrus characteristic of the rat vaginal smear (Table 1), while estrogen replacement induced a prevalence of cornified cells in the rat vaginal smear which represents estrus, as reported by a previous study [25]. At the beginning of the experiment, all rats were 3 months old and weighed at 240~260 g. Three weeks after OVX, rats in all groups accumulated weight (Additional file 7: Figure S6). The rats in OVX group significantly gained weight throughout the course of experiment, and at the end of the experiment, their average weight was significantly higher than that of rats in the control group. The gain in body weight in OVX rats was reversed easily by both modes of estrogen treatment, and at the end of experiment, there was no difference between the E-decrease/increase and the control groups.

**Table 1:** Classification of the estrous cycle of rats in different groups

	Day3	Day21	Day26	Day37	Day41
Control	pro/es/de	pro/es/de	pro/es/de	pro/es/de	pro/es/de
OVX	de	de	de	de	de
E-decrease	de	pre/es	es	es	pre/es
E-increase	de	pre/es	es	es	es

*pro*, proestrus; *de*, di-estrus; *pre*, pre-estrus; *es*, estrus

## Discussion

The present study demonstrated that chronic and continuous estrogen increase or decrease in OVX rats differently affect OVX-induced hypersensitivity in these animals. With our replacement strategies, continuous decline of estrogen at a late stage reversed mechanical hyperalgesia, while progressive elevation of estrogen abrogated its analgesic effect and progressively attenuated mechanical threshold compared with the control group. Coincided with the behavioral changes, chronic estrogen downregulation results in increased ERα and ERβ expression in the spinal cord, while chronic upregulation of estrogen increased spinal ERα more significantly and dramatically reduced ERβ.

It is proposed that the discrepancy regarding estrogen modulation on pain-related behaviors was due to various pain modalities examined and different doses and delivery methods of estrogen used. Based on our and previous results, the inconsistency may be partly explained by chronic estrogen increase or decrease in the animals. Studies investigating rat recognition and memories showed that cyclic rather than continuous estrogen replacement facilitated the acquisition of spatial memory [26]. And chronic estrogen replacement regimes differently affect restoration of cognition [25]. However, the aforementioned studies adopted more than one kind of drug formulations or delivery methods (i.p. injection and pellet implantation), which would inevitably influence the consistency of drug pharmacokinetics among different experimental groups, and thus add another variable to the already complicated mechanisms through which estrogen exert its neuronal modulation effects. In addition, several early studies claimed to obtain sustained serum levels of estrogen with implantation of silastic capsules, yet this method was not adopted widely due to the inefficient drug release from the silastic tube [27]. In the present study, we chose the widely used slow-release estrogen pellets and managed to achieve continuous estrogen increase or decrease trends through careful experimental design; therefore, the approach described here may be superior to those using other drug forms. Furthermore, our pilot study results verified that the widely used estrogen pellets, do provide a consistent release rate in vivo; however, the actual estrogen serum concentration fluctuated dramatically and failed to maintain a steady level, this could be explained by the dynamic metabolism of serum hormones in vivo as well as an accumulation effect in distal tissues.

In summary, the present study is the first to explore the influence of different trends of gradual estrogen change on pain modulation. The most important finding of the present study is that gradual decrease rather than increase of serum estrogen levels was capable of reversing OVX-induced hyperalgesia. Moreover, the mechanical threshold even decreased in the E-increase group to a significantly lower level compared with that of the E-decrease group during the later phase of the experiment, which indicated that continues increasing trend of estrogen level abolished its analgesic effects, and tend to re-establish the mechanical hyperalgesia induced by OVX. These results should enlighten us in that chronic estrogen fluctuation consists of a far more predominant component in pain modulation than was expected, especially under a hypersensitive state in female rats. These results appears to have implications for clinical findings in patients with chronic pain disorders, dynamic decrease in blood estrogen levels have been noted in cases of multiple pain disorders including perimenstrual migraine, and estrogen replacement therapy was associated with risk of orafacial and back pain in women [22]. It remained to be elucidated whether a causal relationship could be established between estrogen increase or decrease trends and modulation of clinical pain-related syndromes in women. Plenty of studies have examined the roles of estrous cycle in somatic and visceral pain modulation [6, 28, 29]. Robbins et al. showed that a sudden estrogen withdrawal achieved by brief estrogen pellet implantation produced a robust increase in VMRs evoked by bladder distension [24], and proestrus-like surge of serum estrogen increased inflammation-evoked nociception in female rats [30]. However, it is intrinsically difficult to generalize the findings based on rat estrus cycle to human. And the short-term alteration of hormone levels was beyond the scope of the present study.

Our results demonstrated that OVX generated persistent mechanical hyperalgesia within 1 week, and a single estrogen pellet replacement was capable of reversing the OVX-induced hypersensitivity. These results were in agreement with previous studies [2, 4, 5], which showed that OVX induced a hyperalgesic state that was deemed as a kind of chronic pain. Thermal nociception was seldom affected as was concluded by Sanoja et al [2]; however, based on our observations, OVX rats showed a trend of decreased thermal pain threshold 3 weeks following the surgery (data not shown). It should be reiterated that the estrogen dose used in our study (2.5 mg, 21-day release pellet) was slightly higher than that in most previous studies; nevertheless, the mechanical hyperalgesia was alleviated dramatically following replacement, which indicates that the estrogenic analgesia effects was dose-independent. In addition, considering that stress levels could also affect animal pain perception, and the multiple blood collection process in our experiments may influence the HPA axis in rats, we also tested the serum corticosterone levels and the grooming duration of rats among different groups during the experiment. Our results are consistent with previous studies in that OVX rats have higher levels of serum corticosterone compared with intact rats [7], and estrogen replacement reduced their stress levels, which may offer some explanation for the nociception difference under different hormone states. However, most importantly, the blood collection process in our experiment has no significant effect on rat corticosterone levels when assessed during the 2-week experiment.

The immunohistochemistry results showed that long-term OVX decreased spinal ERα expression while ERβ was less affected. Previous literatures implicated that the change of ERs may be time-dependent after OVX in rats. For short-term OVX upregulated ERs genes accurately in female rats [14], and long-term OVX gradually downregulated ERs in rat brain [13, 15]. Our observations are in consistent with the studies concerning long-term OVX. Most importantly, our results showed that spinal ERs were differently regulated under the two estrogen replacement regimes as was assessed at the end of the experiment. In the E-decrease group, ERα increased significantly after the 21-day hormone substitution while ERβ was slightly decreased but without significance compared with the control group. These results conflict a previous study, which recorded decrease of both ERs after estrogen replacement [14] . The confliction seems more likely to be a result of different timing of the evaluation of ERs change rather than of different estrogen concentration. Due to the current method limitation, we were unable to monitor the ERs change dynamically throughout the replacement course. On the other hand, in the E-increase group, the expression of spinal ERα was increased even more dramatically, while ERβ was strongly depressed compared with the E-decrease and the control groups. These results indicate that the disparate findings at behavioral level may be linked to the distinctive modulation of central level ERs.

Despite the incoherent results, ERα is mostly associated with classic reproductive-related functions and its role related to pain modulation is conflicting, while ERβ was more predominantly involved in inhibitory effects on nociception in various pain modalities [31, 32], and ablation of either ERα or ERβ would eliminate the sex difference in mechanical nociception in normal and inflamed mice [33]. At the central level, ERα is found to co-localized with preproenkephalin mRNA and estrogen could rapidly increase spinal enkephalin levels [8, 34]. ERα activation was concluded to be antinociceptive in the formalin model [35]; at the same time, it exaggerated visceral pain in several conditions [36]. Khomula et al. proved that ERα agonist facilitates hyperalgesic priming though IP3 receptor by electrophysiological means [37], and ERα antisense attenuated AMP-induced hyperalgesia in primed female rats [38]. These studies collectively confirmed that ERα is involved in central pain pathways. It is possible that the over-activation of ERα further facilitated the hyperalgesia in OVX rats, as was supposed in the E-increase group. Anatomical studies showed ERβ was co-localized with GABAergic neurons through which it may adjust the inhibitory tune at the spinal level [32, 39]. ERβ is also reported to enhance the serotonergic (5-HT) descending inhibitory pathway in rat dorsal raphe nuclei [40]. Together, these results provide fundamental basis for ERβ to exert its analgesic effects during pain transduction. Piu et al. found that ERβ agonists protect from inflammatory pain as well as PEG_2_- or capsaicin-induced hyperalgesia in rats [31, 41–43], and ERβ knockout mice manifested a hyperalgesic phenotype in resemblance of human interstitial cystitis syndromes [44]. Though contradictions exist [35, 45], overall ERβ is capable of alleviating both acute and chronic pain in a relatively ligand-specific manner [46]. We may postulate that the hyperalgesia showed in the E-increase group could be correlated to a depletion of ERβ at the central sites which eliminated its analgesic effects in OVX states.

Despite the multiple signaling pathways implicated in ERs modulation of pain, it is suggested that estrogen’s regulation effects could be deemed as a balance between the two opposing forces derived from the two-receptor subtypes. Induced expression of ERβ is revealed to have bi-directional effects on ERα regulated genes, enhancing or counteracting the effects of ERα in vitro and in vivo [47–49], ERα:ERβ ratio differently affected OVX-induced osteoporotic fracture healing [50]. It is reasonable that the conflicting behavioral results may be attributed to the different expression ratio of central ERα:ERβ accompanying fluctuating levels of estrogen. As has been showed in our results, the disparity in pain responsiveness induced by estrogen was pertinent to different spinal ERα:ERβ expression ratio. It could be anticipated that a mismatch modulatory action of estrogen receptors may lead to a hyperalgesic state as was seen in the E-increase group in the present study.

Estrogen could activate both classical ERs and membrane ERs following activation of its receptors, and both pathways could result in long-term changes in neuronal plasticity and nociception. It has been increasingly recognized that membrane ERs may be more extensively engaged in pain transduction [51]. ERs in brain regions are organized with mGluRs to activate protein kinase A, protein kinase C, and mitogen activated protein kinase (MAPK) cascades [52–54]. However, based on our present observations, it is hard to determine whether estrogen exert its different modulatory effects through which ER-related pathways, and further studies is warranted to elucidate the mechanisms involved.

## Perspectives and significance

The present study investigated the effects of continuous increase or decrease of in vivo estrogen level in a hyperalgesia model of OVX. Our results revealed that estrogen’s nociceptive effect is not only dependent on its presents or not but also on its dynamic change of system level, and this may in some extent explain the contradictions concerning estrogen’s anti-nociception or pro-nociception effects as demonstrated by previous studies; thus, researchers should take into consideration of the effects of estrogen’s dynamic change of level in the future studies.

## Conclusion

The present study extends previous findings concerning the effects of estrogen on nociception. Our results highlight the pivotal role of chronic estrogen increase or decrease in pain modulation from a dynamic point of view. The different mechanism activated by the hormone level change remains to be elucidated.

## Supplementary information

**Additional file 1: Table S1.** Spontaneous behavioral results of rats in different hormone states.

**Additional file 2: Figure S1.** Pharmacokinetic experiments with estrogen pellets. Alterations of serum estrogen concentration when implanted with one single 0.25 mg pellet in rats.

**Additional file 3: Figure S2.** Effects of blood collection on serum corticosterone levels. Change of serum corticosterone levels in rats with different replacement paradigms. The day of OVX surgery was defined as day 0, and arrows indicate the day of estrogen pellets implantation. Corticosterone levels didn’t show significant changes in each group when compared with their own initial concentrations respectively. Data are showed as mean ± SEM.

**Additional file 4: Figure S3.** Effects of physiological level estrogen pellets implantation. (A) Serum estrogen levels when implanted with one single 0.25-mg pellet. (B) Change of PWT in rats of different estrogen treatment groups. Data are shown as mean paw withdraw threshold (± SD) to three times of electronic von Frey stimuli.

**Additional file 5: Figure S4.** Negative control. (A) Immunohistochemistry results of negative control and positive control. (B) Validation of primary antibodies with western blot. ERα and ERβ were detected at molecular weight of 66kd and 55kd respectively.

**Additional file 6: Figure S5.** Low-magnification images show the L4~L6 spinal segments in which all the observations of ERs were conducted. And high-magnification images for differentiate the between the counter stain and the DAB stain. (A) Stain of ERα (B) Stain of ERβ of the L4~L6 spinal segments.

**Additional file 7: Figure S6.** Change of body weight in different groups. Time course of body weight of the rats in one of the following assigned groups: control (black), ovariectomized (blue), E-increase (red), E-decrease (green). Age at the start of the curve was 12 weeks.

## Data Availability

Data sharing not applicable to this article as no data-sets were generated or analyzed during the current study.
