# Supplementary material for: Estrogen modulation of pain perception with a novel 17β-estradiol pretreatment regime in ovariectomized rats
Source: Biol Sex Differ. 2020 Jan 9;11:2. doi: 10.1186/s13293-019-0271-5 (PMC6953313; doi:10.1186/s13293-019-0271-5)
Supplement: Supplementary file 1 — Additional file 1: Table S1. Spontaneous behavioral results of rats in different hormone states. [file 13293_2019_271_MOESM1_ESM.docx]

Table S1. Total duration (d) or frequency (f) of spontaneous behaviors recorded in rats of different hormone states.

| Day 7 after OVX   \| Behavior \| control \| OVX \| OVX+E \| \| --- \| --- \| --- \| --- \| \| Exploration (d) \| 1723±257 \| 1086±123 \| 1578.8±283.4 \| \| Internal crossing (f) \| 174.6±59.4 \| 59±6.8 \| 147.4±28.6 \| \| External crossing (f) \| 598.6±40.5 \| 342±29.7 \| 469±74.3 \| \| Rearing (f)  Grooming (d) \| 134.6±20.8  601.9±136.7 \| 58±6.8  987±135.5 \| 115.7±34.6  651.8±165.4 \| |
| --- | --- | --- | --- | --- | --- | --- | --- | --- | --- | --- | --- | --- | --- | --- | --- | --- | --- | --- | --- | --- |

Data are expressed as mean ±SD.

| Day 14 after OVX   \| Behavior \| control \| OVX \| OVX+E \| \| --- \| --- \| --- \| --- \| \| Exploration (d) \| 1895±291 \| 1129±183 \| 1693±293.2 \| \| Internal crossing (f) \| 165.6±48.5 \| 83±7.4 \| 162.6±35.3 \| \| External crossing (f) \| 573.5±37.5 \| 372±58.3 \| 519±84.3 \| \| Rearing (f)  Grooming (d) \| 120.5±35.2  638.0±143.2 \| 82±6.3  1032±153.3 \| 104.3±28.4  704.3±173.4 \| |
| --- | --- | --- | --- | --- | --- | --- | --- | --- | --- | --- | --- | --- | --- | --- | --- | --- | --- | --- | --- | --- |

Data are expressed as mean ±SD.

| Day 28 after OVX   \| Behavior \| control \| OVX \| OVX+E \| \| --- \| --- \| --- \| --- \| \| Exploration (d) \| 1692±314 \| 1057±193 \| 1593±268.5 \| \| Internal crossing (f) \| 187.3±52.3 \| 93±7.9 \| 157.3±45.2 \| \| External crossing (f) \| 610.3±38.3 \| 385±62.8 \| 586.8±98.4 \| \| Rearing (f)  Grooming (d) \| 209.4±29.8  610.6±184.6 \| 92±9.3  1104±182.3 \| 114.6±36.9  736.6±184.2 \| |
| --- | --- | --- | --- | --- | --- | --- | --- | --- | --- | --- | --- | --- | --- | --- | --- | --- | --- | --- | --- | --- |

Data are expressed as mean ±SD.

| Day 35 after OVX   \| Behavior \| control \| OVX \| OVX+E \| \| --- \| --- \| --- \| --- \| \| Exploration (d) \| 1902±328 \| 1047±137 \| 1785±262.4 \| \| Internal crossing (f) \| 138.4±52.7 \| 92.6±11.4 \| 197.2±36.5 \| \| External crossing (f) \| 519.3±84.3 \| 410±67.8 \| 420.6±73.6 \| \| Rearing (f)  Grooming (d) \| 110.3±25.7  610.2±184.2 \| 65±4.7  993.2±152.6 \| 98.3±21.5  682.5±183.2 \| |
| --- | --- | --- | --- | --- | --- | --- | --- | --- | --- | --- | --- | --- | --- | --- | --- | --- | --- | --- | --- | --- |

Data are expressed as mean ±SD.
