# Supplementary figures and images for: Estrogen modulation of pain perception with a novel 17β-estradiol pretreatment regime in ovariectomized rats
Source: Biol Sex Differ. 2020 Jan 9;11:2. doi: 10.1186/s13293-019-0271-5 (PMC6953313; doi:10.1186/s13293-019-0271-5)

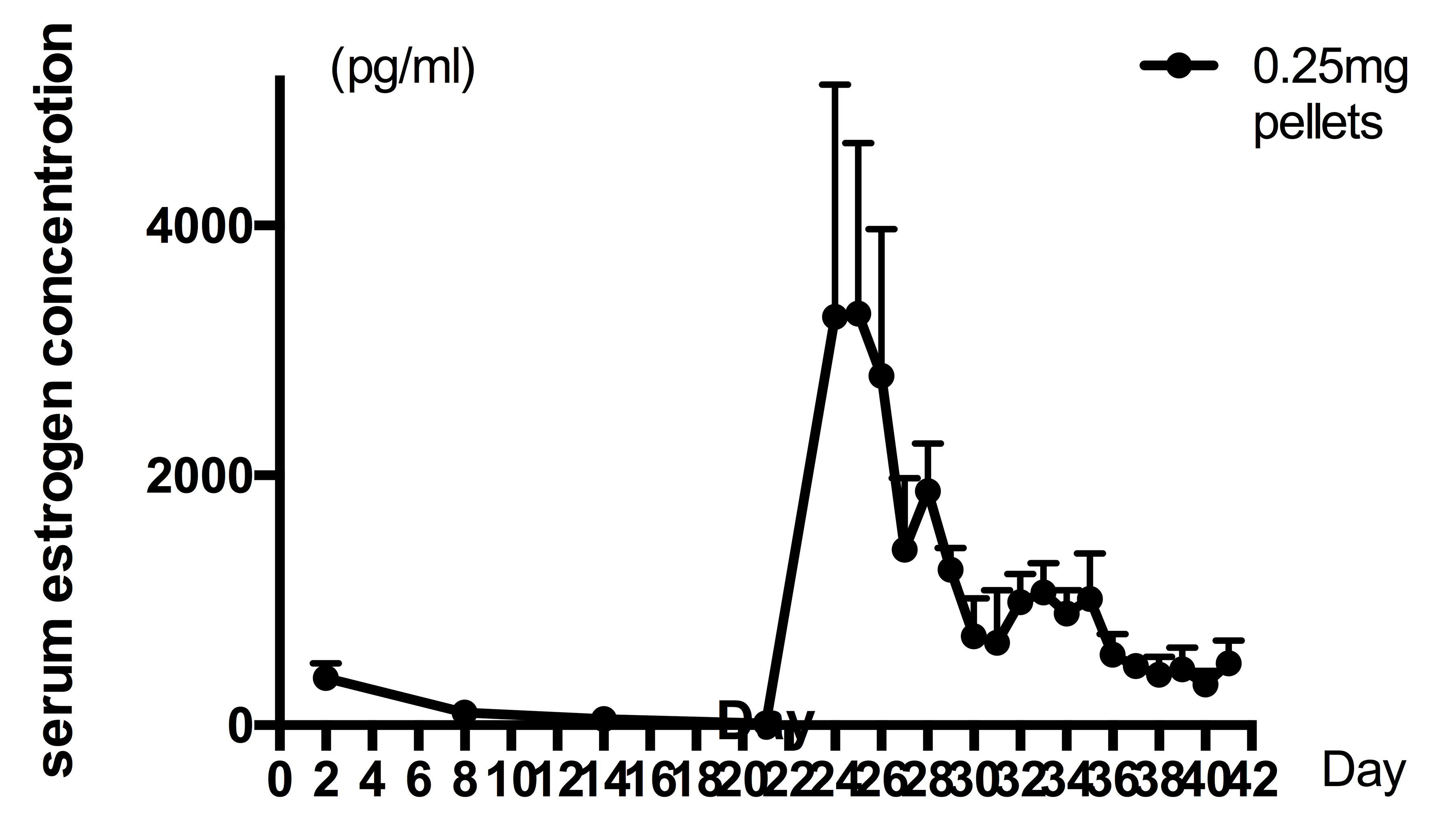

Supplement: Supplementary file 2 — Additional file 2: Figure S1. Pharmacokinetic experiments with estrogen pellets. Alterations of serum estrogen concentration when implanted with one single 0.25 mg pellet in rats. [file 13293_2019_271_MOESM2_ESM.tiff]

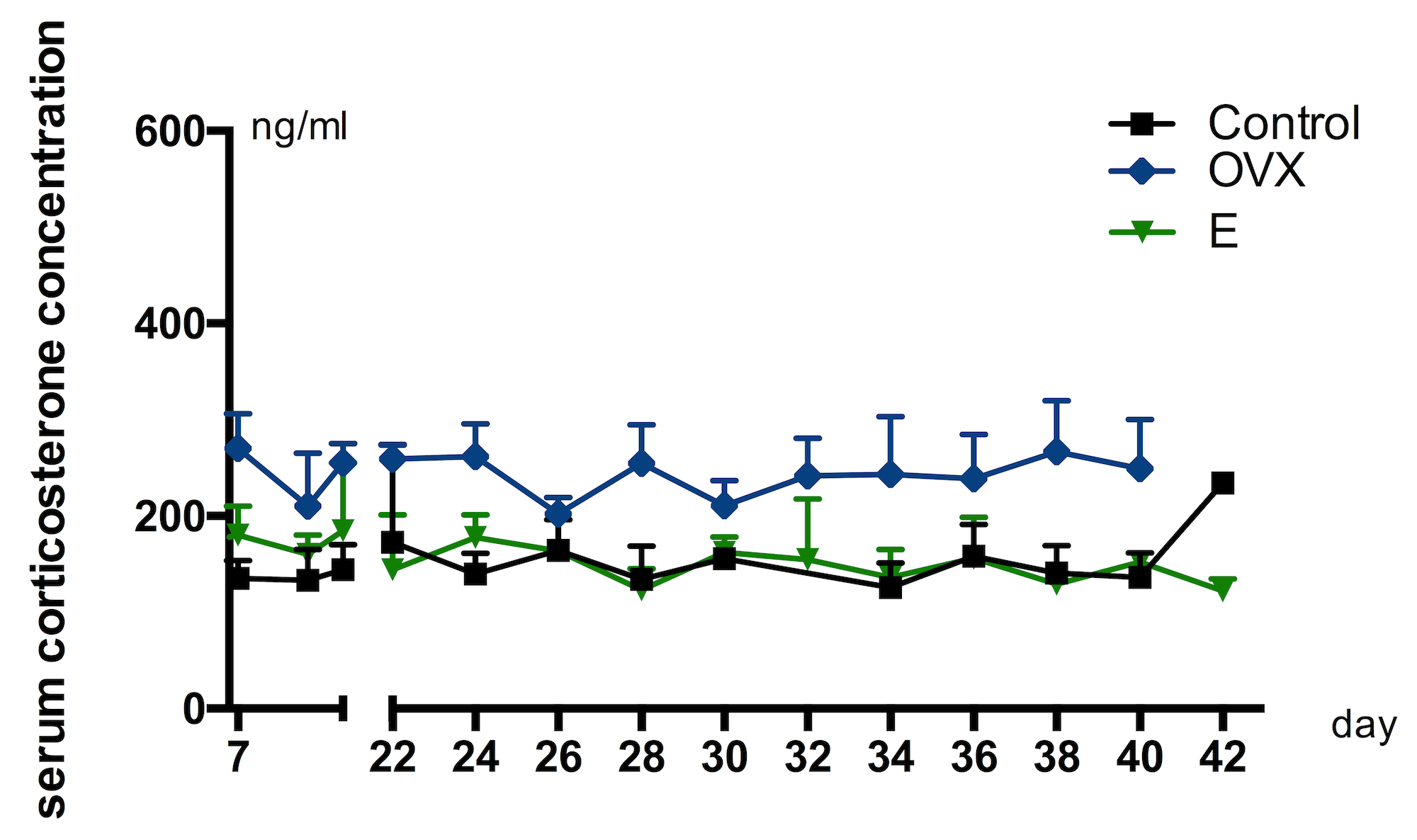

Supplement: Supplementary file 3 — Additional file 3: Figure S2. Effects of blood collection on serum corticosterone levels. Change of serum corticosterone levels in rats with different replacement paradigms. The day of OVX surgery was defined as day 0, and arrows indicate the day of estrogen pellets implantation. Corticosterone levels didn’t show significant changes in each group when compared with their own initial concentrations respectively. Data are showed as mean ± SEM. [file 13293_2019_271_MOESM3_ESM.tiff]

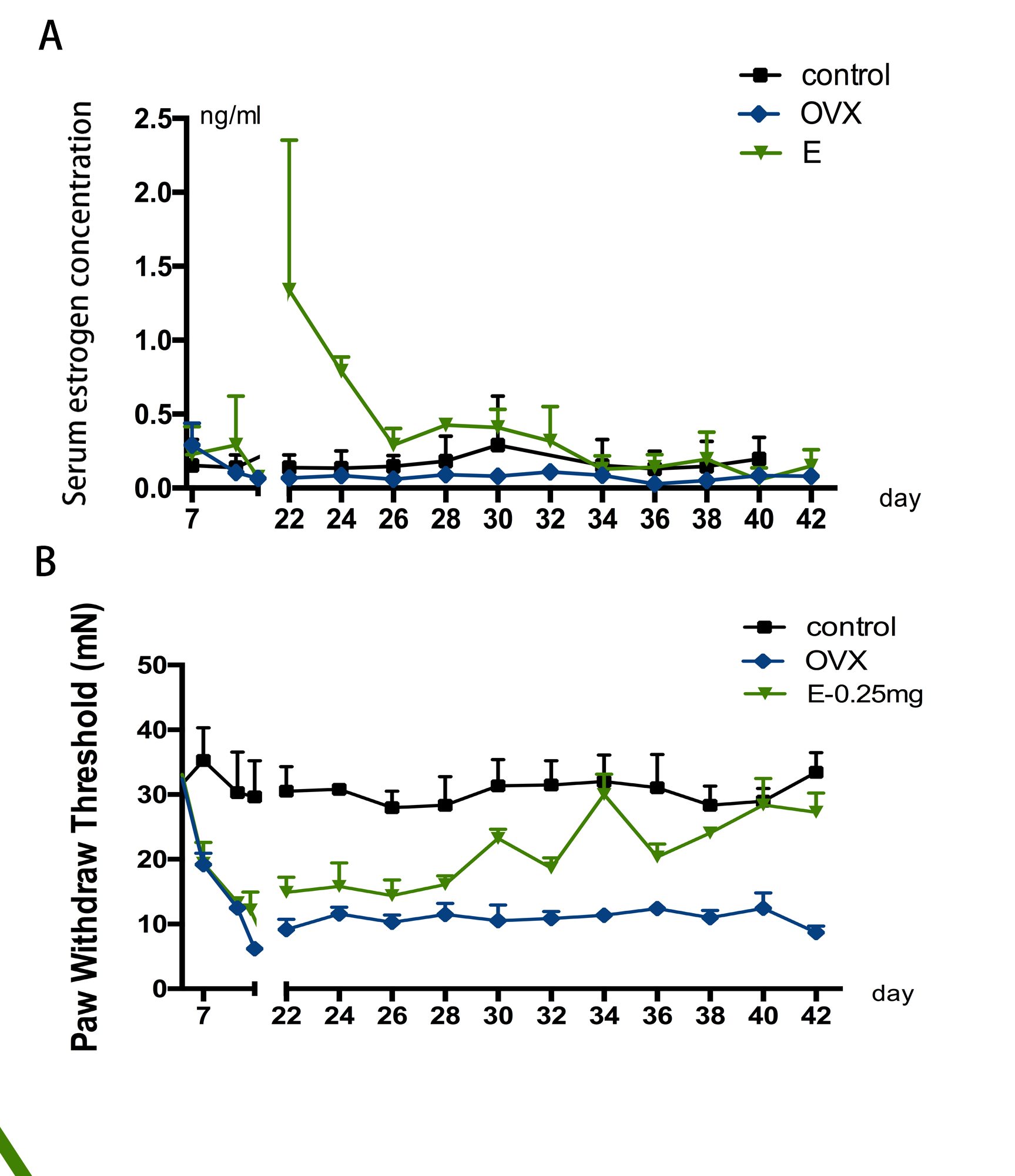

Supplement: Supplementary file 4 — Additional file 4: Figure S3. Effects of physiological level estrogen pellets implantation. (A) Serum estrogen levels when implanted with one single 0.25-mg pellet. (B) Change of PWT in rats of different estrogen treatment groups. Data are shown as mean paw withdraw threshold (± SD) to three times of electronic von Frey stimuli. [file 13293_2019_271_MOESM4_ESM.tiff]

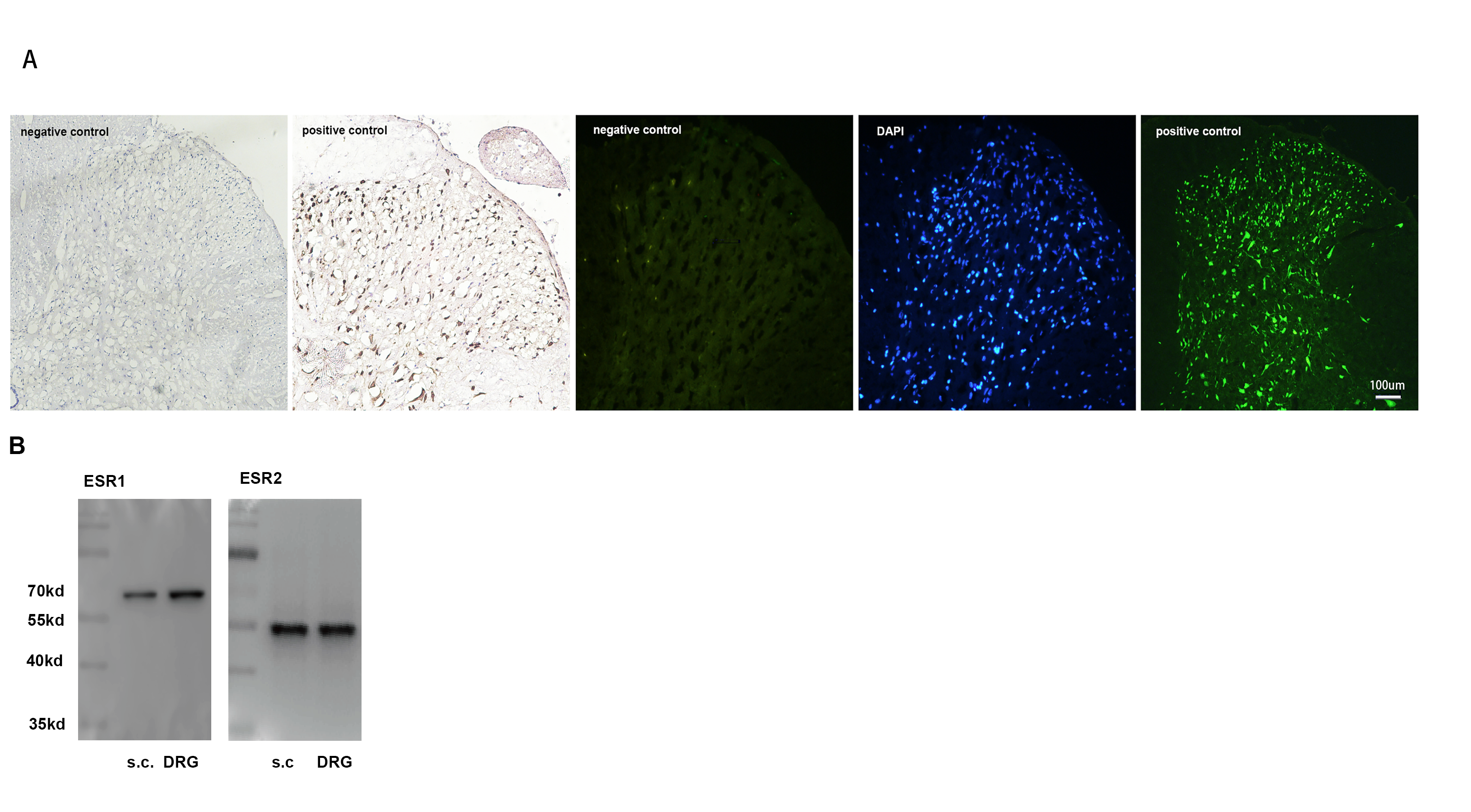

Supplement: Supplementary file 5 — Additional file 5: Figure S4. Negative control. (A) Immunohistochemistry results of negative control and positive control. (B) Validation of primary antibodies with western blot. ERα and ERβ were detected at molecular weight of 66kd and 55kd respectively. [file 13293_2019_271_MOESM5_ESM.tif]

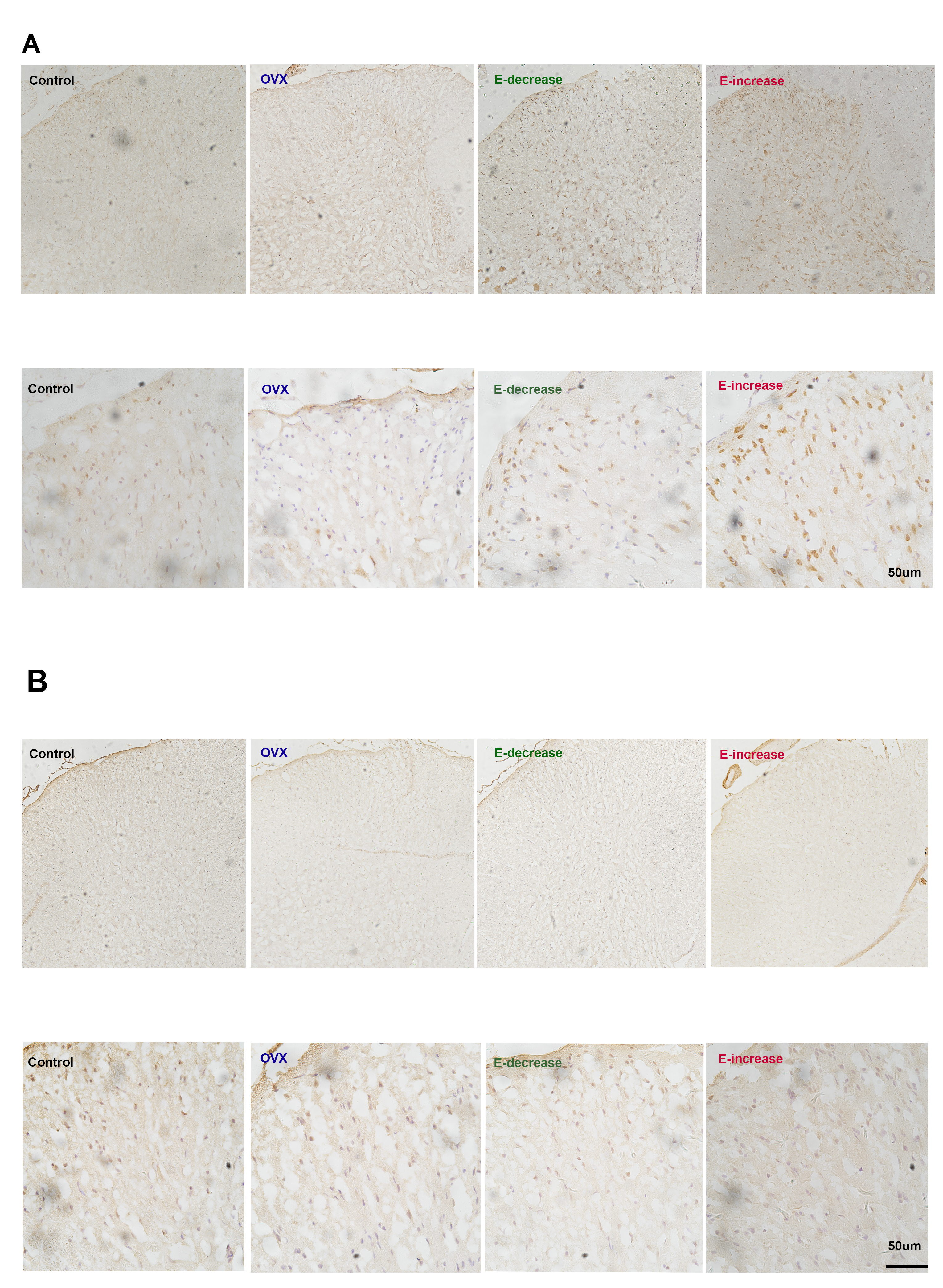

Supplement: Supplementary file 6 — Additional file 6: Figure S5. Low-magnification images show the L4~L6 spinal segments in which all the observations of ERs were conducted. And high-magnification images for differentiate the between the counter stain and the DAB stain. (A) Stain of ERα (B) Stain of ERβ of the L4~L6 spinal segments. [file 13293_2019_271_MOESM6_ESM.tif]

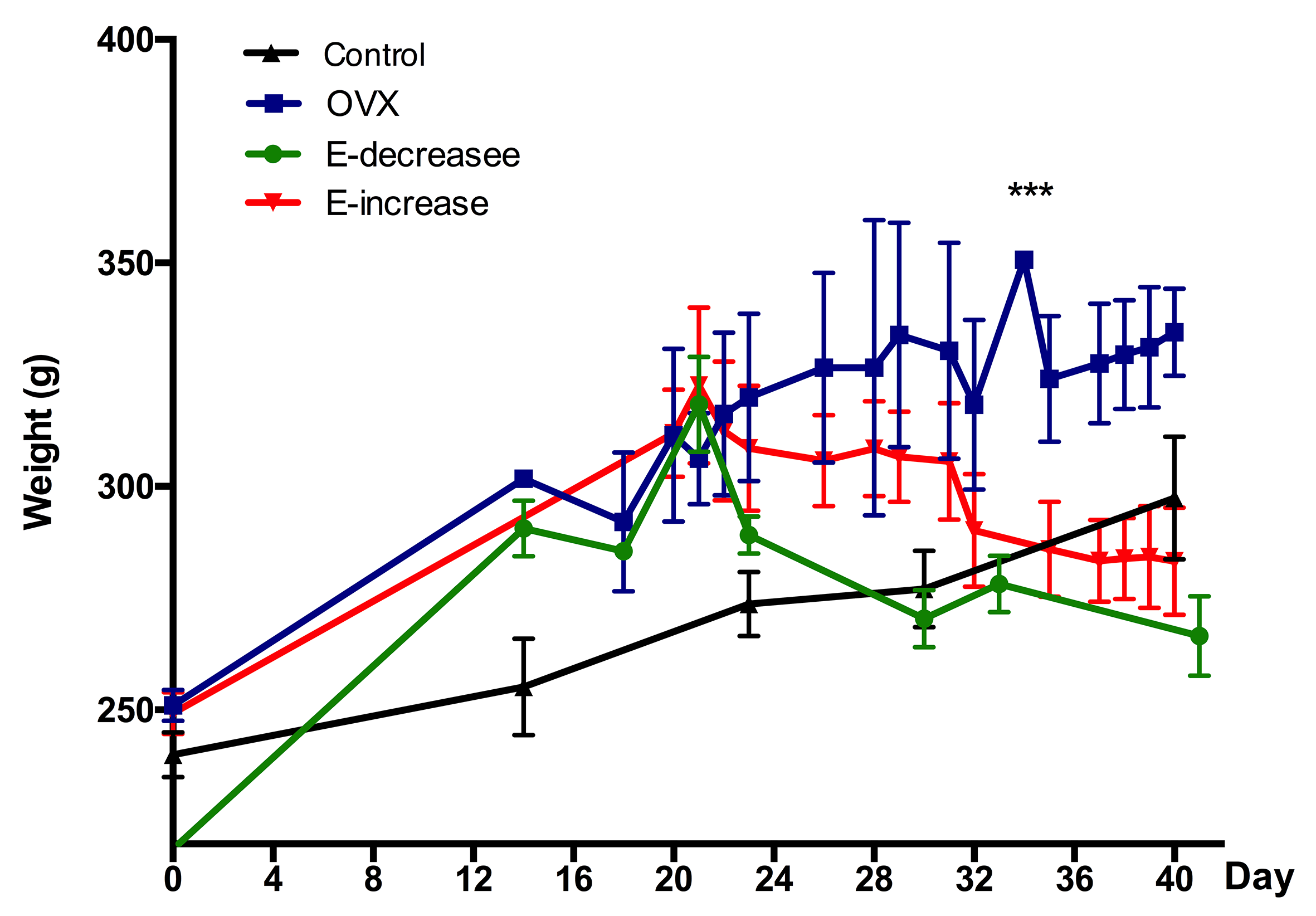

Supplement: Supplementary file 7 — Additional file 7: Figure S6. Change of body weight in different groups. Time course of body weight of the rats in one of the following assigned groups: control (black), ovariectomized (blue), E-increase (red), E-decrease (green). Age at the start of the curve was 12 weeks. [file 13293_2019_271_MOESM7_ESM.tiff]
